# Common Neuropathological changes from Hispanics and Non‐Hispanics enrolled in multiple Alzheimer's Disease Research Centers

**DOI:** 10.1002/alz70855_101342

**Published:** 2025-12-23

**Authors:** Geidy E Serrano, Sidra Aslam, Jessica Walker, Claryssa Borja, Ileana Lorenzini, Monica Mariner, Thomas G Beach

**Affiliations:** ^1^ Banner Sun Health Research Institute, Sun City, AZ, USA; ^2^ Banner Sun Health Research Instittue, Sun City, AZ, USA

## Abstract

**Background:**

Hispanics seem to be at higher risk of developing Alzheimer's disease dementia (ADD) but there are few neuropathologically validated studies.

**Method:**

The National Alzheimer's Coordinating Center (NACC) functions as the centralized data repository for the National Institute of Aging's (NIA's) Alzheimer's Disease Research Centers (ADRC) Program and currently hosts data from over 2,100 Hispanic participants who have donated their brains to the study, and close to 60% of those have had full neuropathological evaluations done at 28 different ADRCs. The main goal of this study is to compare the neuropathological changes observed in these subjects to those reported as non‐Hispanics.

**Result:**

Hispanics in this study self‐identified as Caucasians (72%), African American (4%), Native American (less than 2.5%), or “Other Races or unknown” (20%), as compared to the non‐Hispanics, (82% Caucasian, 14% African American, 0.1% Other). The largest percentage of Hispanics were Mexican or of Mexican descent (64%), followed by 12% Puerto Rican, 6% Cuban, 5% Dominican, 6% South American, with the remainder from other central American countries or Spain. Sex distribution was similar in both groups with close to 49% of the participants identifying their biological sex as males. Hispanic participants died at younger ages (83 vs 84 years), had lower brain weights (1093 +/‐ 152g vs 1116 +/‐ 157g) and had a higher percentage of Intermediate to High levels of AD Neuropathological Changes (75% vs 67%). Vascular pathology was also more severe in Hispanics, both for moderate/severe cerebral amyloid angiopathy (45% to 29%) and moderate/severe cerebral white matter rarefaction (33% to 31%). Hispanics in this data set also had higher rates of heart attack, congestive heart failure, hypertension, and diabetes.

**Conclusion:**

This also agrees with previous clinical studies suggesting that ethnic Hispanic people have greater white matter hyperintensity volumes and higher vascular risk factors, but additional studies are needed to also understand how race intersects ethnicity.

